# Prediction of the molecular action of *Trypanosoma vivax* on bovine reproductive parameters and risk factors associated with trypanosomiasis in northern Minas Gerais, Brazil

**DOI:** 10.14202/vetworld.2025.837-850

**Published:** 2025-04-19

**Authors:** Amanda Cristielly Nunes De Lima, Joely Ferreira Figueiredo Bittar, Otaviano de Souza Pires Neto, Eliane Macedo Sobrinho Santos, Priscilla Elias Ferreira da Silva, Hércules Otacílio Santos, Cintya Neves de Souza, Franciane Gabrielle dos Santos, Anna Christina de Almeida

**Affiliations:** 1 Institute of Agricultural Sciences, Federal University of Minas Gerais, Montes Claros, Minas Gerais, Brazil; 2 Department of Veterinary Medicine, University of Agriculture UNIUBE, Uberaba, Minas Gerais, Brazil; 3 Campus Janaúba, Montes Claros State University, Janaúba, Minas Gerais, Brazil; 4 Campus Araçuaí, Federal Institute of Northern Minas Gerais, Araçuaí, Minas Gerais, Brazil

**Keywords:** bioinformatics, epidemiology, molecular mechanism, reproduction, trypanosomiasis

## Abstract

**Background and Aim:**

Trypanosomiasis caused by *Trypanosoma vivax* is a significant cause of reproductive inefficiency and economic losses in cattle farming. While its impact on reproduction is known, the molecular mechanisms underlying these effects remain poorly understood. This study aimed to investigate the molecular effects of *T. vivax* on reproductive parameters in cattle and evaluate epidemiological risk factors associated with trypanosomiasis in beef cattle in northern Minas Gerais, Brazil.

**Materials and Methods:**

A dual approach combining *in silico* bioinformatics and epidemiological analyses was employed. Proteins linked to *T. vivax* were identified using the UniProt database, and protein interaction networks were constructed using the String V.12 platform. The epidemiological study involved serological diagnosis of trypanosomiasis through indirect immunofluorescence in 383 serum samples collected from 14 herds. Risk factors such as farming system, animal replacement frequency, vector presence, vaccination practices, and reproductive history were assessed through statistical analysis.

**Results:**

Bioinformatics analyses suggested that *T. vivax* may exert molecular effects on bovine reproduction through the expression of toll-like receptor 2, nuclear factor kappa B (NF-κB), and nuclear receptor coactivator 7 proteins. The overall prevalence of *T. vivax* was 6.79%, with no direct association found between infection and reproductive performance. However, 57.7% of seropositive cattle belonged to farms with lower pregnancy rates, and 96% were from farms reporting abortions in the past 12 months. The semi-intensive/intensive farming system and shared use of syringes and needles during vaccination were identified as significant risk factors for *T. vivax* infection.

**Conclusion:**

The study provides evidence of *T. vivax* spread in northern Minas Gerais and highlights the need for improved control strategies, including vector management and proper sanitary practices. Bioinformatic analysis suggests that *T. vivax* may influence reproductive outcomes through the NF-κB signaling pathway, warranting further experimental validation. Future studies should investigate the molecular mechanisms of *T. vivax* in high-prevalence herds to refine disease management and mitigation strategies.

## INTRODUCTION

Trypanosomiasis, the most pathogenic etiological agent of cattle, is *Trypanosoma vivax* [1] and is transmitted cyclically by the fly *Glossina* spp. In Latin America, transmission occurs mechanically, directly from one mammal to another through hematophagous dipterans of the genera *Tabanus* (“horseflies”), *Stomoxys*, and *Haematobia*. The disease has a high economic impact on different productive livestock systems [2–5].

The presence of *T. vivax* in cattle has already been reported in different countries [6–15], including Brazil, with the occurrence of several outbreaks [16–27]. The increase in the vector population during the rainy season and the acquisition of contaminated animals are risk factors for outbreaks of the disease [19, 28–31]. Published and unpublished data on the occurrence of trypanosomiasis outbreaks in different countries are relatively common. However, epidemiological factors linked to the host, environment, and animal management, which favor the occurrence and severity of the infection, are still scarce in the literature [32]. *T. vivax* is a hemoparasite that causes an important reproductive disease of domestic ruminants and is mainly responsible for subfertility, causing economic losses for the producer [19, 33, 34]. Reproductive disorders, including abortion, interruption of the estrous cycle, retained placenta, and perinatal mortality, can be severe, especially when they occur during the last third of pregnancy [32, 35].

Despite extensive documentation on the epidemiology and economic consequences of *T. vivax* infection in cattle, there is a lack of comprehensive understanding of the molecular mechanisms by which this pathogen affects reproductive parameters. Although previous studies have associated *T. vivax* infection with reproductive inefficiencies, such as abortion, estrous cycle disruptions, and perinatal mortality, the specific molecular pathways involved in these outcomes remain poorly elucidated. Furthermore, there is limited data integrating bioinformatics approaches with epidemiological analyses to provide mechanistic insights into the disease’s reproductive impact.

This study aims to investigate the molecular mechanisms through which *T. vivax* influences reproductive parameters in cattle by employing bioinformatics analysis and epidemiological data. Additionally, the study seeks to identify key epidemiological risk factors associated with *T. vivax* infection in beef cattle in northern Minas Gerais, Brazil, to enhance disease management and mitigation strategies.

## MATERIALS AND METHODS

### Ethical approval

This study was approved by the ethics committee for the use of animals at the Federal University of Minas Gerais (process number 201/2020). Only animals whose owners provided consent were recruited for the study. All animals were treated under the National Council for the Control of Animal Experimentation guidelines with the help of a veterinarian.

### Study period and location

The study was conducted from November 2020 to February 2021.

**Figure 1:**
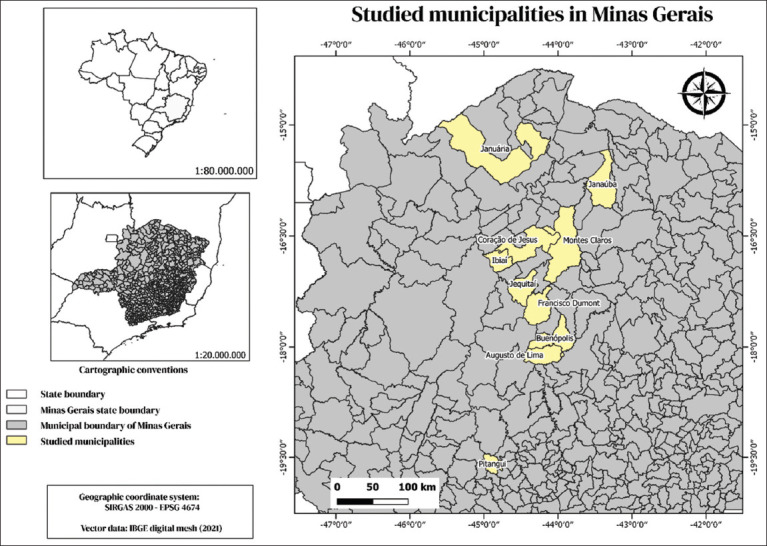
Map of Brazil highlighting the state of Minas Gerais and the 10 municipalities where the 383 animals were surveyed, distributed over 14 rural properties.

The study area is in the northern mesoregion of Minas Gerais (Figure 1). The mesoregion is a part of the Brazilian semi-arid region, also known as the “Drought Polygon.” It has a predominantly humid tropical climate, with dry winters and rainy summers. The average temperature of the coldest month is over 18°C, and the rainfall of the driest month is less than 60 mm. This climate type is predominant in lower altitude areas. It fits into the Köppen-Geiger climate classification as a humid tropical climate (megathermic) [36].

### Experimental design and sampling

**Figure 2:**
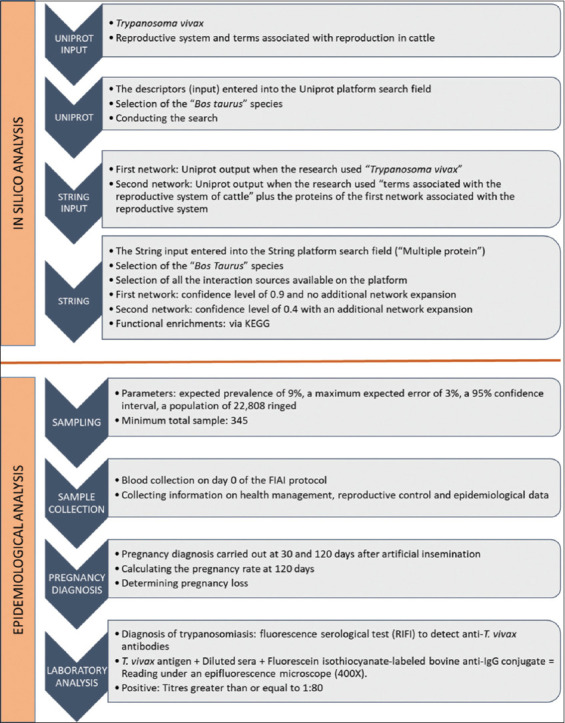
Flowchart showing the methodological steps: *In silico* and epidemiological analyses.

The study was conducted in two stages: *In silico* and epidemiological analyses, as summarized in Figure 2.

The study was epidemiological and cross-sectional, with non-probabilistic convenience sampling. The sample size was calculated according to the recommendations of Thrusfield *et al*. [37]. With an expected prevalence of 9%, according to a recent study carried out by Batista *et al*. [38], a maximum expected error of 3%, a 95% confidence interval (CI), and a population of 22,808 ringed females in the database of the animal reproduction companies that are partners in the research, and a minimum total sample of 345 samples was determined. We surveyed 383 animals, distributed across 14 properties in 10 municipalities in the northern region of Minas Gerais (Figure 1).

### Sample collection and pregnancy diagnosis

A single blood sample was collected from reproductive-age females undergoing the fixed artificial insemination protocol on day 0, and the period corresponding to the reproductive season on the properties evaluated. Five mL of blood was collected from each animal through veni puncture of the coccygeal vein in tubes without anticoagulant (Vacuette^®^, Greiner Bio-One Brasil Ltda, Brazil). The serum was then obtained and stored at –20°C until serology was performed.

The breeding season varied according to the management of each farm (between November and April). Information on health management and reproductive control was analyzed along with epidemiological data. Pregnancy was diagnosed 30 and 120 days after artificial insemination using transrectal ultrasound. The final pregnancy rate was calculated as the number of pregnant cows at 120 days multiplied by 100, divided by the number of animals that were challenged to reproduce [39].

The gestational loss was considered to involve cows that were confirmed to be pregnant for the first time around 30–50 days after insemination and subsequently showed visual signs of abortion or were empty during pregnancy reconfirmation on the 120^th^ day after artificial insemination [40].

### Laboratory analysis

The fluorescence serological test (IIFR - Indirect Immunofluorescence reaction) was performed to detect anti-*T. vivax* antibodies, according to the methodology described by Cuglovici *et al*. [41]. At the time of the reaction, slides containing the *T. vivax* antigen were removed from the freezer and dried at 37°C. The sera were diluted, distributed on slides containing the antigen, and incubated for 30 min at 37°C. Fluorescein isothiocyanate-labeled bovine anti-IgG conjugate (Sigma, USA, Anti-Bovine IgG (whole molecule)–FITC antibody produced in rabbit – F7887) was added at the dilution recommended by the manufacturer in Evans blue and incubated in an oven at 37°C. The slides were read under an epifluorescence microscope (Nikon Eclipse E200^®^, Model UN2-PSU100, Japan) at 400× magnification. Fluorescence reactions with a titer of >1:80 were considered positive [42]. Sera from experimentally infected and uninfected cattle with *T. vivax* were used as positive and negative controls, respectively, at a dilution of 1:80.

### Bioinformatic analysis to predict molecular mechanisms

The analysis began with a search for proteins associated with *T. vivax* in the *Bos taurus* species. This was done using the UniProt digital platform (https://www.uniprot.org/) using “*Trypanosoma vivax*” as the keyword, selecting the organism defined as “*Bos taurus*.” Another search was then carried out on the UniProt platform using the descriptor “Reproductive system” and other terms related to the reproductive system to select the species “*Bos taurus*.”

The output proteins from the UniProt platform were used as input proteins in the String digital platform version 12.0 (https://string-db.org/). On this platform, two protein interaction networks were built by selecting all the interaction sources available on the platform, with a confidence level of 0.9 and no additional network expansion for the first network and a confidence level of 0.4 with an additional network expansion for the second network. A search on the UniProt platform using the descriptor “*Trypanosoma vivax*” yielded the protein used to build the first protein interaction network. To build a second protein interaction network, proteins from the first network with the potential to act on the reproductive system of cattle were used. In addition to these proteins, we used a protein extracted from the UniProt platform when using terms related to bovine reproduction.

The functional enrichments of the elaborated network were obtained through Kyoto Encyclopedia of Genes and Genomes (KEGG), a tool from the String V.12 platform (STRING Consortium 2024, https://string-db.org/).

### Statistical analysis

The following variables were considered in the assessment of risk factors: Breeding system (semi-intensive/intensive or extensive), type of activity (breeding or full cycle), frequency of animal replacement (6 months to 1 year or 2 years or more), presence of flooded areas accessible to animals (yes or no), presence of insect vectors (yes or no), implementation of horn fly control measures (yes or no), shared use of syringes and needles (yes or no), record of abortions in the past 12 months (yes or no), use of reproductive vaccines (yes or no), vaccination of all females for brucellosis (yes or no), performance of brucellosis diagnostic tests in the herd (yes or no), and acquisition of males or females for reproduction (yes or no).

The data were analyzed using descriptive statistics in the Statistical Package for the Social Sciences (SPSS), version 23 (IBM SPSS Statistics for Windows, Armonk, NY, USA). To evaluate associations between categorical variables, Pearson’s Chi-square test (χ²) was applied at a significance level of 5% (p < 0.05). In cases where expected values were less than five, Fisher’s exact test was used. Odds ratios (OR) and 95% CIs were also calculated to quantify the strength of associations between risk factors and *T. vivax* infection.

For the subgroup of animals that tested positive for *T. vivax*, Student’s t-test was employed to assess potential relationships between infection status and key epidemiological variables considered important for disease transmission and prevalence.

## RESULTS

Of the 383 animals evaluated, 26 were seropositive for protozoans, corresponding to a prevalence of 6.79%. Of the 14 properties sampled, 8 had at least one animal that was seroreactive in the RIFI test. No *T. vivax*-positive animals were found in 4 of the 10 municipalities of origin in Minas Gerais, Brazil: Montes Claros, Francisco Dumont, Januária, Augusto de Lima, Buenópolis, Jequitaí, Ibiai, Janaúba, Pitangui, and Coração de Jesus.

Among the epidemiological variables subjected to statistical analysis, the variables “farming system” related to the properties and “vaccination practice,” especially against reproductive diseases, were significantly associated with the occurrence of trypanosomiasis. The semi-intensive/intensive system carries a greater risk of seropositivity than the extensive system. Regarding reproductive information, the use of shared syringes and needles in vaccination practice was associated with a greater risk of trypanosomiasis (Table 1).

**Table 1:** Risk factors associated or not with *T. vivax* infection in cattle from the northern mesoregion of Minas Gerais. with the respective OR, CI, and statistical significance.

Epidemiological variables	*T. vivax* (%)		OR	95% CI	p-value

	Positive	Negative			
Breeding System			0.377	0.143–0.796	0.009*
Semi-intensive/Intensive	18 (10.5)	154 (89.5)			
Extensive	8 (3.8)	203 (96.2)			
Type of activity			1.299	0.502–3.358	0.373
Create	20 (6.4)	290 (93.6)			
Full cycle	6 (8.2)	67 (91.8)			
Purchase of animals for breeding			-	-	-
Yes	26 (6.8)	357 (93.2)			
No	0	0			
Frequency of animal replacement			0.913	0.884–0.943	0.103
6 months to 1 year	26 (7.4)	326 (92.6)			
2 years or more	0	31 (100)			
Sharing needles			-	-	-
Yes	26 (6.8)	357 (93.2)			
No	0	00eww			
Presence of flooded areas			1.172	0.524–2.622	0.43
Yes	11 (6.3)	165 (93.8)			
No	15 (7.2)	192 (92.8)			
Presence of vectors			1.515	0.584–3.934	0.267
Yes	20 (6.3)	298 (93.7)			
No	6 (9.2)	59 (90.8)			
Horn fly control			-	-	-
Yes	26 (6.8)	357 (93.2)			
No	0	0			
Record of abortion in the last 12 months			0.285	0.038–2.153	0.163
Yes	25 (7.4)	313 (92.6)			
No	1 (2.2)	44 (97.8)			
Use of reproductive vaccines			0.178	0.052–0.604	0.001*
Yes	23 (10.0)	206 (90.0)			
No	3 (1.9)	151 (98.1)			
Brucellosis vaccine for females			-	-	-
Yes	26 (6.8)	357 (93.2)			
No	0	0			
Brucellosis test			-	-	-
Yes	0	0			
No	26 (6.8)	357 (93.2)			

* Statistical difference (p < 0.05). - No statistics were calculated because the parameter is constant. OR=Odds ratio, CI=Confidence interval, *T. vivax=Trypanosoma vivax*

**Figure 3:**
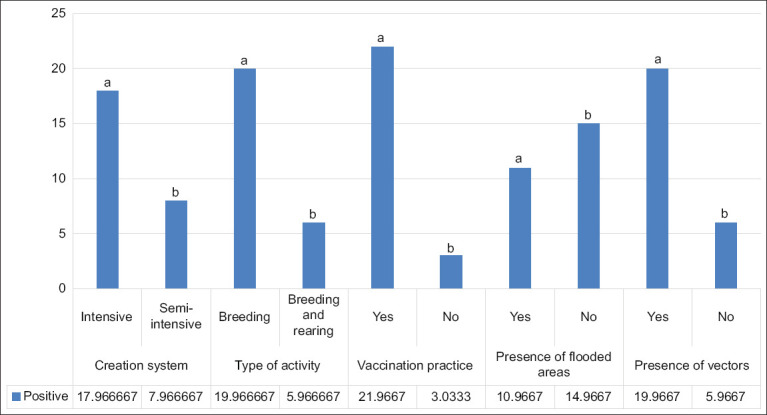
Quantification of seropositive animals under different risk factor conditions for *Trypanosoma vivax* infection in cattle in the northern Mesoreodon of Minas Gerais. ^a,b^The letters indicate statistically different means according to Student’s t-test at 5% significance (p < 0.05).

Corroborating the previous data, we found that the number of positive animals was higher on farms that kept animals in an intensive or semi-intensive system (p = 0.000). Similarly, the number of animals affected by *T. vivax* was higher in herds where the practice of reproductive vaccinations using shared syringes and needles was reported in the questionnaires (p = 0.000) (Figure 3).

We also found that 76% of the animals seropositive for *T. vivax* came from a type of incomplete cycle breeding (only calves) (p = 0.000). As expected, most of the positive animals (76.9%) were owners who reported the presence of insect vectors on their properties (p = 0.000). However, the highest number of seropositive animals (57.7%) for *T. vivax* was observed in properties where the animals had no access to flooded areas (p = 0.000) (Figure 3).

Analyses of reproductive data, such as the pregnancy rate at the induced assisted timed fertilization (IATF), final pregnancy rate, and gestational loss on the farms evaluated, showed no association with the prevalence rate of trypanosomiasis (Table 2). Thus, the presence of *T. vivax* did not interfere with the reproductive indices of the farms examined in this study.

**Table 2:** Reproductive indices of the farms evaluated in relation to seroprevalence for *T. vivax* during the observation period.

Reproductive rates	*T. vivax*		

	Positive	Negative	p-value
IATF pregnancy rate	52.79 ± 2.38	51.01 ± 3.11	0.531
Final pregnancy rate	76. 10 ± 1.44	78.28 ± 4.35	0.072
Pregnancy loss	6.06 ± 1.25	6.64 ± 0.94	0.739

Statistical difference (p < 0.05). Values are shown in mean ± standard error. *T. vivax=Trypanosoma vivax*

**Figure 4:**
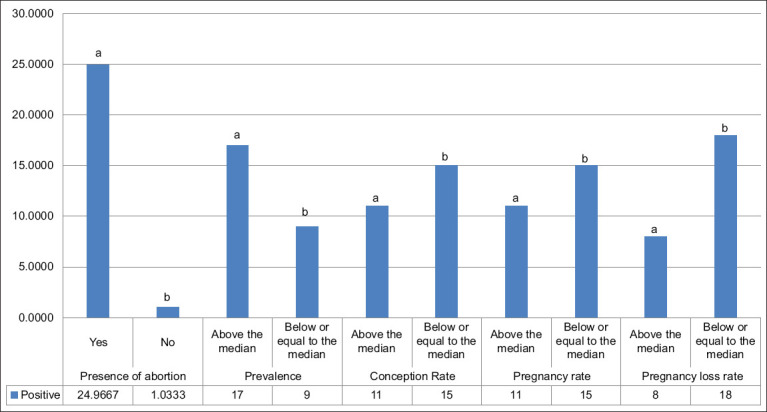
Number of animals seropositive for *Trypanosoma vivax* according to reproductive data from cattle in the northern Mesoreodon of Minas Gerais. ^a,b^The letters indicate statistically different means using the Student’s t-test at 5% significance (p < 0.05).

Figure 4 shows that 57.7% of the animals seropositive for *T. vivax* came from farms that had a pregnancy rate at the IATF and a final pregnancy rate below or equal to the median of the group of seropositive animals (p = 0.000). In addition, 96% of the animals with a positive diagnosis came from farms that had confirmed abortions in the past 12 months (p = 0.000). However, regarding the rate of pregnancy loss, only 30% of the animals diagnosed with trypanosomiasis belonged to properties with a loss rate above the median.

These results help understand the role of general health and reproductive management practices in the epidemiology of trypanosomiasis in cattle in northern Minas Gerais, Brazil. The low prevalence of trypanosomiasis in this study may have contributed to the lack of association between the disease and some risk factors. Therefore, bioinformatic analysis was designed to increase the evidence of the relationship between trypanosomiasis and reproductive impacts in cattle farming, thereby making the results more conclusive and robust.

The toll-like receptor 2 (TLR2) and nuclear receptor coactivator 7 (NCOA7) proteins were extracted from the UniProt platform using descriptors related to *T. vivax* and the bovine reproductive system, respectively. These proteins were used in the first and second protein interaction networks, respectively. The NCOA7 protein was selected owing to its action on estrogen, which plays a role in various reproductive events.

**Figure 5:**
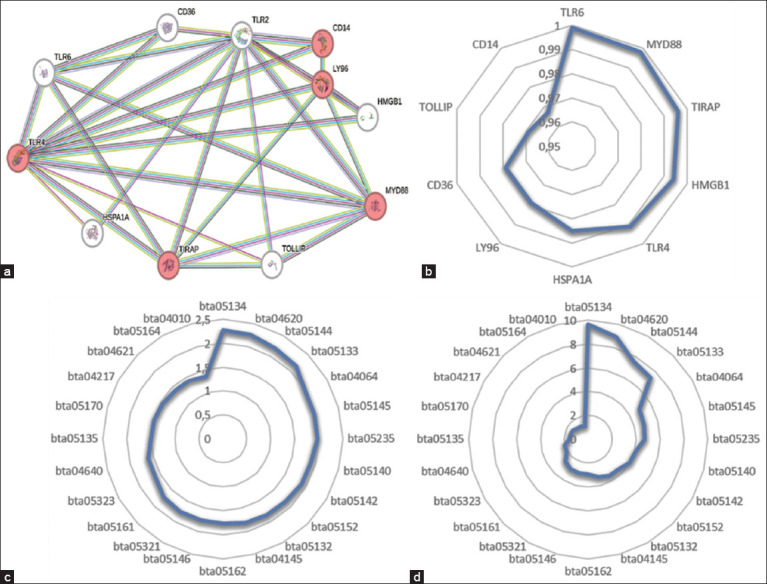
Parameters involved in the protein interaction network constructed using the toll-like receptor 2 protein in the *Bos taurus* species on the String V.12 platform. (a) Interaction network showing proteins with potential action in the reproductive system (colored in red). (b) Confidence score assigned to each protein according to the quantity and quality of the links between the proteins in the network. (c) Analysis of the Kyoto Encyclopedia of Genes and Genomes (KEGG) pathway, showing the strength of the signaling pathway in the network. (d) KEGG pathway analysis showing the percentage of proteins from a pathway that are part of the network.

The bioinformatic analysis conducted to generate a protein interaction network based on the TLR2 input protein revealed a network that included at least 5 proteins (colored in red) that could alter reproductive parameters in cattle (Figure 5a). As can be seen in Figure 5b, the confidence score for the interconnections between the proteins in the network was >0.9, indicating a high degree of interconnection between the proteins in the network. Analysis of the CD14 (monocyte differentiation antigen CD14), LY96 (lymphocyte antigen 96), MYD88 (myeloid differentiation primary response protein MyD88), TIRAP (TIR domain containing adaptor protein), and TLR4 (toll-like receptor 4) proteins showed that they are part of an important signaling pathway in the reproductive system, the NF-kB pathway (bta04064). Figures 5c and d show the analysis of the KEGG pathway, showing the strength of a signaling pathway in the network (Figure 5c) and the percentage of proteins from a pathway that are part of the network (Figure 5d). The NF-kB signaling pathway exerts a force of 1.99 on the network, with the highest force observed at 2.28. In addition, it is clear that of the 101 proteins involved in the NF-kB signaling pathway, 5 proteins were included in the network developed in this study, pointing to a possible molecular mechanism between *T. vivax* and the reproductive aspects of cattle.

**Figure 6:**
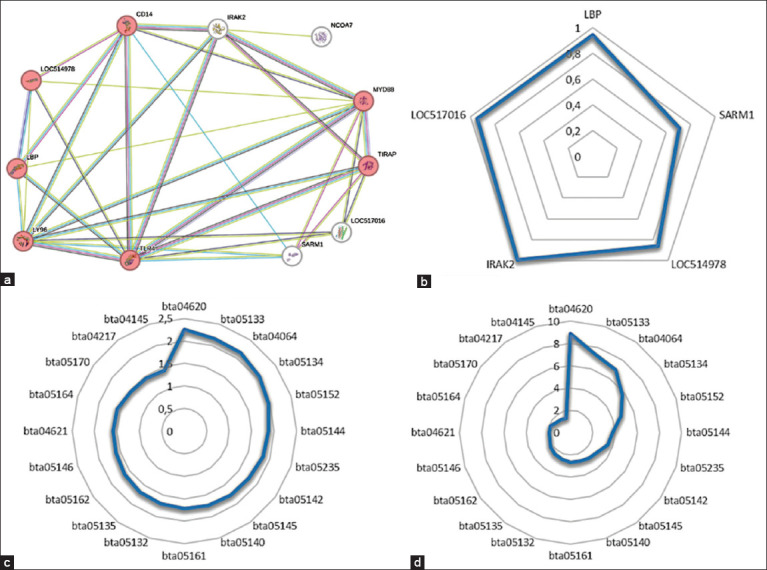
Parameters involved in the protein interaction network constructed using the nuclear receptor coactivator 7, CD14 (monocyte differentiation antigen CD14), LY96 (lymphocyte antigen 96), MYD88 (myeloid differentiation primary response protein MyD88), TIRAP (TIR domain-containing adaptor protein), and toll-like receptor 4 proteins in *Bos taurus* on the String V.12 platform. (a) Interaction network showing proteins with potential action in the reproductive system (colored in red). (b) Confidence score is assigned to each protein according to the quantity and quality of the links between the proteins in the network. (c) Analysis of the Kyoto Encyclopedia of Genes and Genomes (KEGG) pathwayshowsg the strength of the signaling pathway in the network. (d) KEGG pathway analysis shows the percentage of proteins from a pathway that is part of the network.

In the second protein interaction network developed through bioinformatic analysis to predict the molecular mechanisms involved in the action of *T. vivax* on reproductive parameters in cattle, the input proteins NCOA7, CD14, LY96, MYD88, TIRAP, and TLR4 were used. It is possible to see the interconnection of NCOA7 in the network (Figure 6a), where the lowest binding score was 0.708 (Figure 6b). The network proteins indicated in red have a network insertion strength of 2.14 (Figure 6c) and are involved in the NF-kB signaling pathway (7 out of 101 proteins - Figure 6d).

## DISCUSSION

Determining the prevalence of important trypanosomes within a geographical region is essential for understanding the epidemiology and behavior of the disease. In this study in the northern region of Minas Gerais, Brazil, the involvement of 6.79% of the animals and 57.14% of the properties by *T. vivax* indicated the expansion of the disease in Brazil, given that in a previous study in the same region, the prevalence of bovine trypanosomosis was 1.38% [43]. Despite the increase in prevalence in the study region, it is still a lower prevalence than those recorded in other semi-arid regions of Brazil, where prevalence reached 33.8%, 29.7%, and 25.4% in cattle, goats, and sheep, respectively [44, 45].

In Minas Gerais, Brazil, trypanosomiasis usually occurs acutely, with fever, anorexia, weight loss, reduced productivity, neurological alterations, abortion, and perinatal mortality [18, 46, 47]. Factors such as low efficiency of mechanical transmission, animal resistance, and low virulence of *T. vivax* isolates contribute to the occurrence of subclinical trypanosomiasis. In endemic areas, this form of the disease favors the production of antibodies that protect cattle affected by the parasite [44]. In the chronic phase of the infection, the animals become asymptomatic, making diagnosis difficult and leading to the animals becoming carriers/reservoirs of the infection, thus spreading the disease [44, 48].

Among the risk factors for the prevalence of trypanosomiasis, the farming system adopted by the properties was associated with the occurrence of the disease, and the semi-intensive/intensive system offered a greater risk of seropositivity when compared to the extensive system (OR: 0.377; 95% CI: 0.143–0.796) (Table 1). The heterogeneity of livestock farming systems and their marked regional specificity trigger the existence of problems of different types, especially those related to herd health issues [49]. Intensified systems favor the occurrence of pathogenic microorganisms on farms [50].

When analyzing risk factors in relation to trypanosomiasis in cattle in Venezuela, Suárez *et al* [51] found that on farms with semi-intensive management, the cattle had higher levels of active infection (6.3%) and seropositivity (33.6%) than those detected in cattle under extensive management (5 and 26.5%, respectively). These differences were significant only for seropositivity. Confirming these results, there was a positive association between semi-intensive management and seropositivity (OR = 1.40), with this variable being a risk factor, whereas extensive management had a protective effect (OR = 0.71).

In relation to the use of reproductive vaccines using shared syringes and needles, there was a higher risk of trypanosomiasis (OR: 0.178; 95% CI: 0.052–0.604) (Table 1). This is associated with the sanitary practices adopted by producers because poor sanitary practices, such as using the same needle for several animals during the administration of medicines and vaccines, can help spread *T. vivax* [22, 38]. Similarly, when analyzing the risk factors associated with *T. vivax* infection, Meneses [43] indicated a greater chance of the disease occurring in herds in which the owner declared having been tested for brucellosis. Several studies have reported the use of oxytocin and/or vaccines as predisposing factors for the occurrence of trypanosomosis in the respective herds [52, 53].

The relationship observed between infection by *T. vivax* and the characterization of the breeding activity, with the activity characterized by the breeding phase showing a greater number of positive animals, may be related to the greater age of the females in a breeding system. Suárez *et al* [51] stated that the adult age of the animals represented greater exposure to risk for both active infection (OR = 2.14) and seropositivity (OR = 1.41), compared to animals <1 year old.

When analyzing only seropositive animals, this study showed an association between seropositivity to *T. vivax* and the presence of insect vectors, such as tabanids, *Stomoxys* spp., and *Haematobia irritans*. Studies have analyzed the risk factors for trypanosomiasis in cattle point to an increase in the number of cases in rainy periods, when the density of insect vectors increases [38, 54–56].

The act of foraging in flooded areas did not contribute to the increase in positivity. However, these results are inconsistent with data in the literature [38]. In flooded areas, humidity and temperature contribute to the production of forage plants and the proliferation of hematophagous insect vectors of the disease [38]. These differences in the studies can be attributed to various factors, including differences in vegetation types and seasons when the studies were conducted; these factors are known to affect insect populations and, ultimately, the prevalence of trypanosome infections. Therefore, more studies are needed to evaluate the transmission capacity of *T. vivax* in flooded areas.

In relation to the interviewees’ reports of abortions on the properties evaluated, 96% of the positive animals came from properties that had confirmed abortions in the past 12 months. There is evidence of vertical (transplacental) transmission of *T. vivax* in cattle and sheep [57], suggesting that this route of transmission is associated with the occurrence of abortions, prematurity, intrauterine growth retardation, and perinatal mortality [58, 59]. A seroprevalence of 50% (51/102) has been reported for *T. vivax* in cattle with reproductive disorders. The protozoan is currently associated with abortions in different Brazilian regions [60].

When comparing the data on pregnancy rate at IATF, final pregnancy rate, and gestational loss between the farms identified as positive and negative for *T. vivax*, no statistical difference was observed for any of the variables analyzed (Table 2). However, this does not mean that *T. vivax* infection does not affect conception and pregnancy in cattle, and it is necessary to evaluate these parameters on farms with a higher prevalence of the disease. When comparing reproductive efficiency data for uninfected and infected cows collected before and after a *T. vivax* outbreak on a dairy farm, Batista *et al*. [38] suggested that infection reduced reproductive rates, and the effects persisted even in the absence of parasitemia [22].

The literature points to a decrease in the pregnancy rate at IATF and final pregnancy rate in the group of positive animals, showing that the cows in the infected group had a significant delay in the onset of the first postpartum estrus and an increase in the number of estrus occurrences, a longer service period, an increase in the calving interval, and higher abortion rates [38, 61].

Therefore, elucidating the molecular mechanisms that limit and regulate reproductive processes in cattle is fundamental to defining timely diagnosis, prevention, and treatment. The results obtained from the bioinformatics analysis suggest the molecular action of *T. vivax* in the reproductive system of cattle (Figures 5 and 6). The TLR2 protein, used as an input descriptor for the elaboration of the protein interaction network, is located on the cell surface and recognizes components of the pathogen membrane and appears to induce the production not of IL-12, but of TNF-α and NO [62]. In the protein interaction network constructed in this study, TLR2 appears to be interconnected with 5 proteins that are part of the NF-kB signaling pathway.

The NF-kB transcriptional factor is a nuclear factor that participates in both physiological and pathological processes. This transcriptional factor may be involved in the control of inflammatory processes, immune response, stress response, apoptotic processes, growth factors, and enzymes, modulating various biological and pathological processes [63, 64]. In reproductive processes, the NF-kB signaling pathway participates in pregnancy phenomena, such as normal and premature birth [65–67].

As a result, bioinformatic analysis was used to demonstrate the interaction between the NF-kB signaling pathway proteins and the NCOA7 protein (Figure 6a). This protein, also known as estrogen receptor activating protein ERAP140, binds and responds to estrogen receptor α (ERα) and other nuclear receptors, acting as a transcriptional activator [68]. Estrogen plays a central role in the control of sexual behavior and reproductive functions, embryonic and fetal development, and cardiovascular and neuronal physiology [69, 70].

**Figure 7:**
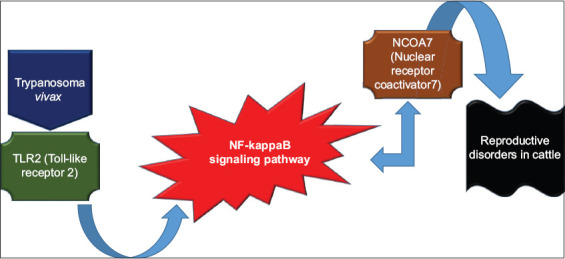
Hypothesis of the molecular mechanism of action of *Trypanosoma vivax* on bovine reproductive parameters.

In summary, the bioinformatic analyses conducted in this study allow us to hypothesize a molecular pathway for *T. vivax* in the reproductive system of cattle. The pathway starts with the TLR2 protein modulating the NF-kB signaling pathway, which in turn acts on the Ncoa7 protein, triggering disturbances in the reproductive system of the animals (Figure 7).

## CONCLUSION

This study provides critical insights into the epidemiology and potential molecular mechanisms of *T. vivax* infection in cattle in northern Minas Gerais, Brazil. The findings highlight an increasing prevalence of *T. vivax* in the region, reinforcing the need for more effective disease control strategies. Epidemiological analysis identified key risk factors, including semi-intensive/intensive farming systems and shared syringes and needles, significantly contributing to disease transmission. Moreover, while no direct statistical association was found between *T. vivax* infection and reproductive parameters, a high proportion of seropositive animals originated from farms with lower pregnancy rates and higher abortion occurrences, suggesting an indirect impact on reproductive health.

The bioinformatics analysis further strengthens the hypothesis of a molecular mechanism linking *T. vivax* infection to reproductive disturbances through the NF-κB signaling pathway. This suggests a potential interaction between pathogen-associated molecular patterns and host immune responses that could disrupt reproductive functions. These findings contribute to a growing body of evidence on the reproductive consequences of bovine trypanosomiasis and underscore the importance of molecular approaches in understanding host-pathogen interactions.

A key strength of this study is its integrative approach, combining epidemiological data with *in silico* bioinformatics analyses to provide both field-based and mechanistic insights into *T. vivax* infection. The robust statistical analysis further validates the association of specific risk factors with disease occurrence, offering valuable information for disease management strategies. However, some limitations must be acknowledged. The relatively low prevalence of *T. vivax* in the sampled population may have limited the statistical power to detect significant associations with reproductive parameters. In addition, *in silico* predictions of molecular interactions require experimental validation to confirm the hypothesized pathways. Moreover, the study was confined to a specific geographical region, which may limit the generalizability of the findings to other cattle-rearing regions with different environmental and management conditions.

Future research should focus on longitudinal studies with larger sample sizes to establish a clearer causal relationship between *T. vivax* infection and reproductive inefficiencies. In addition, experimental validation of the NF-κB signaling pathway’s role in *T. vivax*-induced reproductive dysfunction is necessary to confirm the molecular mechanisms proposed by this study. Developing targeted intervention strategies, such as improved vaccination protocols, vector control measures, and enhanced farm biosecurity, could further mitigate the economic and reproductive impacts of the disease. Finally, expanding the study to other regions and production systems would provide a broader epidemiological perspective on bovine trypanosomiasis in Brazil and beyond. By addressing these gaps, future research can contribute to a comprehensive understanding of the interplay between *T. vivax* infection, cattle reproductive health, and disease transmission dynamics, ultimately leading to more effective control and prevention strategies in endemic regions.

## AUTHORS’ CONTRIBUTIONS

ACDA, JFFB, and ACNDL: Study design and conception. EMSS, PEFDS, and HOS: Drafted the manuscript, data analysis, laboratory analysis, and bioinformatics analysis. FGDS, CNDS, and EMSS: Literature review, statistical analysis, and manuscript formatting. ACNDL and ODSPN: Collected samples and data. All authors have read, reviewed, and approved the final manuscript.
